# Supplementary material for: Anti-HCV antibody titer highly predicts HCV viremia in patients with hepatitis B virus dual-infection
Source: PLoS One. 2021 Jul 1;16(7):e0254028. doi: 10.1371/journal.pone.0254028 (PMC8248640; doi:10.1371/journal.pone.0254028)
Supplement: S1 Fig — A. All patients. B. HCV mono-infected patients. C. HBV dually infected patients. (PDF) [file pone.0254028.s001.pdf]

S1 Fig. AUROC of the anti-HCV titer in predicting HCV RNA seropositivity.

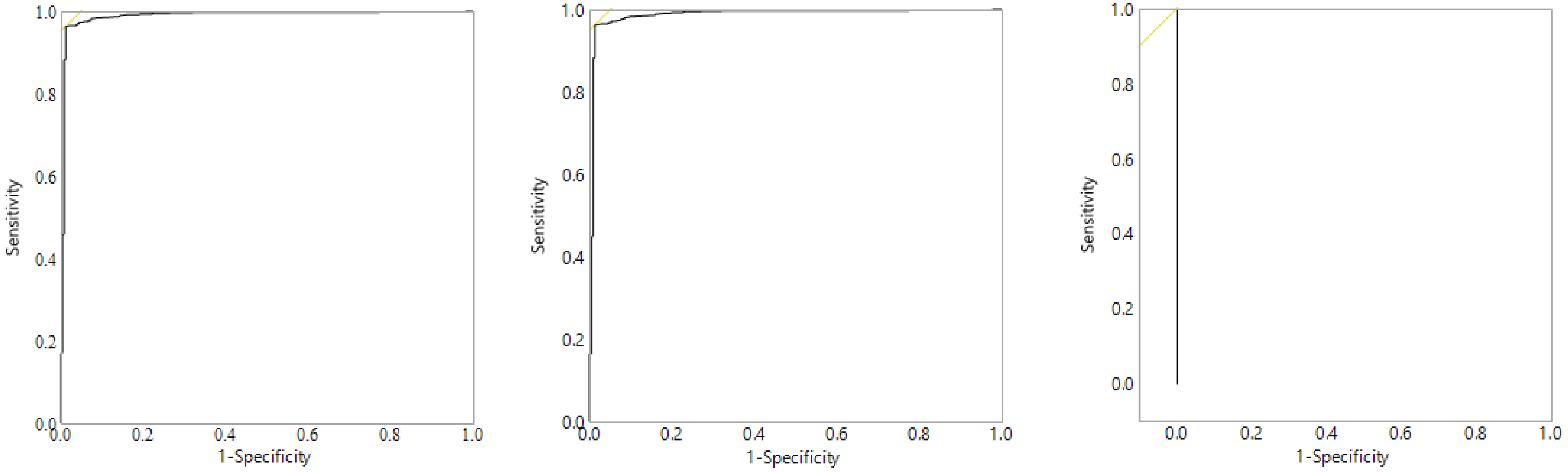

(A). All patients, the best cut-off value of anti-HCV titer in predicting HCV viremia was 9.95 (AUROC 0.99, P<0.0001)

(B). HCV mono-infected patients, the best cut-off value of anti-HCV titer in predicting HCV viremia was 9.95 (AUROC 0.99, P<0.0001)

(C). HBV dually infected patients, the best cut-off value of anti-HCV titer in predicting HCV viremia was 9.36 (AUROC 1.00, P<0.0001)
