# Supplementary material for: Anti-HCV antibody titer highly predicts HCV viremia in patients with hepatitis B virus dual-infection
Source: PLoS One. 2021 Jul 1;16(7):e0254028. doi: 10.1371/journal.pone.0254028 (PMC8248640; doi:10.1371/journal.pone.0254028)
Supplement: S1 Table — (DOCX) [file pone.0254028.s002.docx]

S1 Table. Comparison of patients whose anti-HCV S/CO <5 but HCVRNA (+) and their counterpart patients.

|  | Anti-HCV <5 S/CO but HCVRNA (+), n=3 | Anti-HCV <5 S/CO and HCV RNA (-), n=199 | P value |
| --- | --- | --- | --- |
| Age, years, mean (SD) | 68.0 (2.0) | 59.1 (14.5) | 0.0009* |
| Male gender, n (%) | 2 (66.7) | 96 (48.2) | 0.53 |
| HCV Genotype 1, n (%) | 0 | 0 | 0 |
| HCV Non-genotype 1, n (%) | 3 (100) | 0 | 0 |
| AST, IU/L (median, range) | 32 (17-108) | 26 (11.2-1258) | 0.85 |
| ALT, IU/L (median, range) | 34 (18-84) | 24 (7.4-932) | 1.00 |
| GGT, IU/L (median, range) | 116 (84-148) | 22 (8-1311) | 0.36 |
| HCV RNA log IU/mL (mean, SD) | 3.03 (0.99) | - | - |
| HBs Ag (+), n (%) | 0 | 35 (17.6%) | 0.42 |
| HBV DNA log IU/ml (mean, SD) | - | 4.3 (1.9) | - |

Note: HCV: hepatitis C virus. AST: aspartate aminotransferase. ALT: alanine aminotransferase. GGT, gamma-glutamyl transferase. HBsAg: hepatitis B surface antigen.
