# Supplementary material for: Anti-HCV antibody titer highly predicts HCV viremia in patients with hepatitis B virus dual-infection
Source: PLoS One. 2021 Jul 1;16(7):e0254028. doi: 10.1371/journal.pone.0254028 (PMC8248640; doi:10.1371/journal.pone.0254028)
Supplement: S2 Table — (DOCX) [file pone.0254028.s003.docx]

S2 Table. Comparison of patients whose anti-HCV >10 S/CO but HCVRNA (-) and their counterpart patients.

|  | Anti-HCV≧10 S/CO but HCVRNA (-), n=3 | Anti-HCV≧10 S/CO and HCVRNA (+), n=1010 | P value |
| --- | --- | --- | --- |
| Age, years, mean (SD) | 63.7 (12.7) | 63.4 (11.5) | 0.98 |
| Male gender, n (%) | 1 (33.3) | 461 (45.6) | 0.67 |
| HCV Genotype 1, n (%) | 0 | 561 (55.5) | 0 |
| HCV Non-genotype 1, n (%) | 0 | 449 (44.5%) | 0 |
| AST, IU/L (median, range) | 23 (22-28) | 59 (18-479) | <0.0001* |
| ALT, IU/L (median, range) | 16 (13-18) | 70 (12-743) | <0.0001* |
| GGT, IU/L (median, range) | 16 (12-20) | 42 (8-1137) | 0.005* |
| HCV RNA log IU/mL (mean, SD) | - | 5.59 (0.99) | - |
| HBs Ag (+), n (%) | 0 | 76 (7.5%) | 0.62 |
| HBV DNA log IU/ml (mean, SD) | - | 3.14 (1.4) | - |

Note: HCV: hepatitis C virus. AST: aspartate aminotransferase. ALT: alanine aminotransferase. GGT, gamma-glutamyl transferase. HBsAg: hepatitis B surface antigen.
