# Supplementary material for: Anti-HCV antibody titer highly predicts HCV viremia in patients with hepatitis B virus dual-infection
Source: PLoS One. 2021 Jul 1;16(7):e0254028. doi: 10.1371/journal.pone.0254028 (PMC8248640; doi:10.1371/journal.pone.0254028)
Supplement: S3 Table — (DOCX) [file pone.0254028.s004.docx]

S3 Table. Studies of different anti-HCV testing methods and their cut-off values in predicting HCV viremia.

| **Reference** | **Region** | **Patient number, n** | **Method** | **Genotype,**  **n (%)** | **Cut-off value**  **(S/CO)** | **Sensitivity** | **Specificity** | **PPV** | **NPV** |
| --- | --- | --- | --- | --- | --- | --- | --- | --- | --- |
| Fahimeh R et al. [7] | Iran | 265 | ELISA | N/A | 2.7 | 100% | 81.4% | 77.2% | 100% |
| Seo YS et al. [8] | Korea | 487 | CMIA | GT1:83 (46.4)*  GT2:96 (53.6)* | 10.9 | 94.4% | 97.3% | 98.3% | 91.4% |
| Payan C et al. [20] | France | 600,000 | MEIA | N/A | 34 | 100% | 93.3% | 98.1% | 100% |
| Current study | Taiwan | 1321 | CMIA | GT1: 578(55.1)  GT2: (38.2) | 10 | 96.3% | 98.9 % | 99.7% | 87.3 % |

Note: *Data available in 179 patients. CMIA, chemiluminescent microparticle immunoassay. MEIA, microparticle enzyme immunoassay. ELISA, enzyme-linked immunosorbent assay. GT: genotype. N/A: not available. PPV: positive predictive value. NPV: negative predictive value.
