# Supplementary material for: Anti-HCV antibody titer highly predicts HCV viremia in patients with hepatitis B virus dual-infection
Source: PLoS One. 2021 Jul 1;16(7):e0254028. doi: 10.1371/journal.pone.0254028 (PMC8248640; doi:10.1371/journal.pone.0254028)
Supplement: Attachment — Submitted filename: Response to Reviewers.docx [file pone.0254028.s005.docx]

Dear Editors,

We greatly appreciate your kind consideration for us to revise our manuscript. Enclosed please find the revised version of our paper **“ Anti-HCV antibody titer highly predicts HCV viremia in patients with hepatitis B virus dual-infection”**. We would like to thank the reviewers for their extensive assessment of our manuscript, and for the important and helpful comments and suggestions. We have taken all the remarks into account, in a manner that is described in detail below together with or responses. We think that, following the reviewers’ suggestions, our manuscript has gained in clarity and hope that the amendments will be considered satisfactory. Below is our detailed list of replies to the reviewers’ comments. Meanwhile, a revised clean copy and a highlighted copy of our manuscript have been submitted to the Manuscript Central.

Thank you very much!

Best regards,

**Chung-Feng Huang M.D., Ph.D.**

Hepatobiliary Division, Department of Internal Medicine, Kaohsiung Medical University Hospital, Kaohsiung, Taiwan

**Tel:**	+886-7-312-1101 ext. 7475

**Fax:**	+886-7-3123955

**Email**: **fengcheerup@gmail.com**

Reviewer #1:

1. Please mention if patients had history of anti-HBV therapy including interferon therapy.

**Reply: Of the 125 HBV dually infected patients, only 3 (2.4 %) underwent nucleoside/nucleotide analogues. Since the case number was limited, it shall not have impact on the result regarding the issue. We have added it in the result of the revised manuscript. Thank you.**

2. As for patients with dual-infection, did all have HBV DNA positive? Please mention if there were any patient who had HBV DNA negative. Is there difference in HCV viral load between mono-infected and dual-infected patients?

**Reply: Thank you for the comment. Of the 125 HBV dually infected patients, forty-nine (39.2 %) had detectable HBV DNA (lower detection limit: 20 IU/mL). The HCV RNA level did not differ between patients with HCV monoinfection and HBV dual infection (5.6 log IU/mL vs. 5.5 log IU/mL, P=0.42). We have denoted it in the revised table 1 accordingly.**

3. Please mention whether all patients with HCV viremia were confirmed to have persistent infection.

**Reply: Thank you for the great comment. The study was executed in the outpatient departments of the medical center. We only tested HCV RNA once for patients who were seropositive for anti-HCV. Most of the patients who were confirmed HCV viremia would be referred and allocated to antiviral therapy. We did not leave the viremic patients being observed and recheck HCV RNA at least 6 months apart thereafter. As mentioned in the text, we may fail to identify and exclude patients with acute hepatitis C infection. However, all the blood samples were retrieved from outpatient clinics during daily practice where acute hepatitis was less likely to be encountered. We have denoted it as the potential limitation in the current study. Thank you again for the comment.**

4. Please provide HCV RNA viral load according to the anti-HCV antibody titer.

**Reply: Among the 1196 HCV viremic patients, the HCV RNA levels were 3.0 log IU/mL, 5.4 log IU/mL, 5.6 log IU/mL and 5.6 log IU/mL in patients whose anti-HCV antibody titer were < 5 S/CO, 5-10 S/CO, 10-15 S/CO and > 15 S/CO, respectively (trend P=0.13). We have denoted it in the revised manuscript.**

5. Please mention to what extent the different method of anti-HCV measurement affects the anti-HCV titer and cut-off value according to the previous reports.

**Reply: Thank you very much for the insightful comment. We fully agree with the review that different anti-HCV testing methods (ex. ELISA, CMIA or MEIA) may result in different cut-off values and accuracy in predicting HCV viremia. We have summarized previous reports and the current study in the supplementary Table 3. Notably, the current study was in line with the report by Seo et al [1]. who used the same chemiluminescent microparticle immunoassay and resulted in a similar cut-off value and accuracy.**

6. In the discussion, please elaborate factors affecting low anti-HCV titer with HCV viremia or high anti-HCV titer with HCV negativity.

**Reply: Thank you for the great comment. As shown in the original Table 3, patients with the extreme mismatch between anti-HCV titer and viremic status were rare. We further analyzed the characteristics of the patients. As shown in Supplementary Table 1, patients (n=3) with anti-HCV S/CO <5 but HCVRNA (+) were older than the counterpart patients. By contrast, patients (n=3) with anti-HCV >10 S/CO but HCVRNA (-) had lower levels of aspartate aminotransferase, alanine aminotransferase and gamma-glutamyl transferase compared to the counterpart patients (supplementary Table 2). We have denoted in the revised manuscript. Thank you again.**

7. The method of HCV genotyping is not described.

**Reply: HCV genotyping was measured using a standardized automated qualitative reverse transcription-polymerase chain reaction (RT-PCR) assay (Abbott Real-Time HCV Assay in m2000 PCR System). We have denoted it accordingly.**

Reviewer #2: In the manuscript titled “ Anti-HCV antibody titer highly predicts HCV viremia in patients with hepatitis B virus dual-infection”, the authors showed the valuable titer to predict Hepatitis C viremia in patiets with HCV mono-infection.
The authors defined HCV infection as HCV-Ab positive. I think that the definition of patients with HBV infection should include not only HBsAg positive but also HBsAb or HBcAb positive. There are patients whose HBsAg are negative but HBV-DNA are positive. HBsAg negative cannot exclude HBV infection.

**Reply: Thank you much for the comment. The study was executed in the outpatient departments. We did not routinely check HBcAb and HBsAb for each subject, and it would be also difficult for us the check HBV DNA in HBsAg-/HBcAb+ subjects. As a general rule, HBV infection is defined as HBsAg seropositivity in the clinical setting, which we adopted the definition in the current study. We fully concur with the reviewer that albeit the patient number should be rare, patients with occult HBV infection (OBI, HBsAg-/AntiHBc+/HBV DNA+) were not identified. We have denoted it as the limitation in the current study. Thank you again for your great comment.**

**Reference**

1. Seo YS, Jung ES, Kim JH, Jung YK, Kim JH, An H, Yim HJ, Yeon JE, Byun KS, Kim CD, Ryu HS, Um SH. 2009. Significance of anti-HCV signal-to-cutoff ratio in predicting hepatitis C viremia. Korean J Intern Med 24:302-8.
