# Supplementary material for: Anti-HCV antibody titer highly predicts HCV viremia in patients with hepatitis B virus dual-infection
Source: PLoS One. 2021 Jul 1;16(7):e0254028. doi: 10.1371/journal.pone.0254028 (PMC8248640; doi:10.1371/journal.pone.0254028)
Supplement: Attachment — Submitted filename: Response to Reviewers.docx [file pone.0254028.s006.docx]

Dear Editors,

We greatly appreciate your kind consideration for us to revise our manuscript. Enclosed please find the revised version of our paper **“ Anti-HCV antibody titer highly predicts HCV viremia in patients with hepatitis B virus dual-infection”**. We would like to thank the reviewers for their extensive assessment of our manuscript, and for the important and helpful comments and suggestions. We have taken all the remarks into account, in a manner that is described in detail below together with or responses. We think that, following the reviewers’ suggestions, our manuscript has gained in clarity and hope that the amendments will be considered satisfactory. Below is our detailed list of replies to the reviewers’ comments. Meanwhile, a revised clean copy and a highlighted copy of our manuscript have been submitted to the Manuscript Central.

Thank you very much!

Best regards,

**Chung-Feng Huang M.D., Ph.D.**

Hepatobiliary Division, Department of Internal Medicine, Kaohsiung Medical University Hospital, Kaohsiung, Taiwan

**Tel:**	+886-7-312-1101 ext. 7475

**Fax:**	+886-7-3123955

**Email**: **fengcheerup@gmail.com**

Reviewer #1: According to table 1, most HBsAg positive patients did not have detectable HBV DNA level. I wonder if such infection stage frequently occurs in the natural course of HBV infection. How about HBsAg level in these patients?

**Reply: We regret that HBsAg level was not available in the retrospective study. As mentioned in the discussion, there may exist reciprocally suppressive effect of the two viruses, and HCV often overwhelms HBV in the same host during the natural course.^1^ The mean HBV DNA level was quite low in the present survey, which was similar to our previous report.^2^ The rate of detectable HBV DNA was around 40 % in the current study, which was also not far from another community-based^3^ or hospital-based^4^ studies regarding HBV/HCV dual infection (<50 %). Patients were consecutively enrolled in the current study and we believed that there would be minimal selection bias. We have denoted it in the 2^nd^ revised version. Thank you again for the great comment.**

**References**

1.	Raimondo G, Brunetto MR, Pontisso P, et al. Longitudinal evaluation reveals a complex spectrum of virological profiles in hepatitis B virus/hepatitis C virus-coinfected patients. Hepatology 2006;43:100-7.

2.	Huang CF, Dai CY, Lee JJ, et al. Hepatitis C viremia interferes serum hepatitis B virus surface antigen and DNA levels in hepatitis B uremics. Hepatol Int 2014;8:224-232.

3.	Yu ML, Dai CY, Huang CF, et al. High hepatitis B virus surface antigen levels and favorable interleukin 28B genotype predict spontaneous hepatitis C virus clearance in uremic patients. J Hepatol 2014;60:253-9.

4.	Yeh ML, Huang CF, Huang CI, et al. Hepatitis B-related outcomes following direct-acting antiviral therapy in Taiwanese patients with chronic HBV/HCV co-infection. J Hepatol 2020;73:62-71.
